# Protocol for a scoping review on the development of policy, guidelines and protocols within emergency medical services

**DOI:** 10.29045/14784726.2022.03.6.4.48

**Published:** 2022-03-01

**Authors:** John Renshaw, Mary Halter, Tom Quinn

**Affiliations:** Kingston University and St George’s, University of London; University of Wolverhampton ORCID iD: https://orcid.org/0000-0002-5774-5877; Kingston University and St George’s, University of London ORCID iD: https://orcid.org/0000-0001-6636-0621; Kingston University and St George’s, University of London ORCID iD: https://orcid.org/0000-0002-5116-0034

**Keywords:** emergency medical services, guideline, policy, protocol

## Abstract

**Introduction:**

Emergency medical services (EMS) use a combination of policy, clinical practice guidelines and protocols to set out their expectations for service delivery and to inform patient care. While these are integral to how EMS now operate, relatively little is known about how they are developed, or the processes involved. Therefore, the aim of this scoping review is to understand what is known in the literature about the development of policy, guidelines and protocols within EMS.

**Methods:**

This scoping review will follow the Arksey and O’Malley (2005) methodological framework for scoping reviews. A search strategy has been developed using index term definitions, building from authors’ knowledge of the field. The following electronic databases will be searched from 2002 to 2021 for all types of publication: CINAHL, Medline, Academic Search Complete and PsycINFO, EMBASE, Nursing and Allied Health, the Cochrane library, NICE Evidence, Scopus, OpenGrey, EThOS, Google Scholar, Google search and key EMS journal websites. The results will be downloaded using EndNote^X9^ reference management software and duplicates will be removed. Titles and abstracts of the results will be independently screened for their relevance to the research question, and the full text of each selected publication will be assessed against pre-determined inclusion and exclusion criteria to determine its eligibility. The reference list and forward citations will be searched for articles meeting the eligibility criteria. A second researcher will independently assess a 10% sample of results to allow for validation of this assessment. Data will be extracted and charted on the characteristics of the publications and the knowledge they contribute on the development of policy, guidelines or protocols. Accompanying narratives will be presented to identify themes and gaps in the available evidence. A critical appraisal will be undertaken of the included publications, where empirical research is presented.

## Introduction

Emergency medical services (EMS) are known to use a combination of policy, clinical practice guidelines (CPGs) and protocols to support front-line EMS clinicians with their clinical decision-making and patient care (Brouwers et al., 2010; Brown et al., 2014b). In recent years, the use of policy, guidelines and protocols has played an important role in the implementation of evidence-informed practice within EMS by (a) setting out what action is required to manage common clinical presentations in the pre-hospital setting and (b) describing how these actions should be performed in clinical practice (Brown et al., 2019; Deakin et al., 2015; National Institute for Health and Care Excellence, 2021). Around the world, EMS appears to be moving away from direct medical oversight and protocol-driven care largely based on expert consensus (Brown et al., 2014b; Munk et al., 2009), towards a model of evidence-informed practice (Brown et al., 2014a; Jensen & Dobson, 2011). In the latter, EMS clinicians have greater flexibility to exercise judgement in the use, and contextualisation, of evidence-based CPG, incorporating resource availability, patient choice, the clinical situation and clinical expertise (Brown et al., 2014b; Sackett et al., 1996). Numerous evidence-based policies, guidelines and protocols exist on issues such as safeguarding, acute myocardial infarction (AMI) and the management of out-of-hospital cardiac arrest (OHCA) in the pre-hospital setting (Chew et al., 2016; Link et al., 2015; NHS England, 2019).

Policy, CPG and protocols are used differently within EMS depending on: (a) how an EMS system is structured and organised, (b) the type of care being directed and (c) the size and location of the EMS organisation (Malherbe et al., 2020; Martin-Gill et al., 2016; National Association of EMS Physicians, 2015). Individual EMS organisations typically develop their own policy, guidelines and protocols in order to communicate their expectations for clinical practice and service delivery within the context of the system of care in each region (Brown et al., 2014a, 2014b). In addition, several organisations also publish national CPGs and protocols for EMS practice. As examples, in the United Kingdom, the Joint Royal Colleges Ambulance Liaison Committee (JRCALC) and the Resuscitation Council UK publish national guidelines for EMS clinicians and pre-hospital care (Brown et al., 2019, p. 9; Deakin et al., 2015).

Two recent systematic reviews of evidence-based guidelines (EBGs) in pre-hospital care found that they frequently vary in quality and have noticeable gaps in their methodology and adherence to reporting standards (Fishe et al., 2018; Turner et al., 2020). Even less is known about the development and quality of the individual policies, guidelines and protocols that guide clinical practice and service delivery within the confines of each EMS organisation (Coppola et al., 2020). Hence, there is a need to better understand the process involved in the development of these documents within EMS to avoid unwarranted variation in EMS clinical practice. The purpose of this scoping review is to identify and map the existing evidence on the development of policy, guidelines and treatment protocols within EMS to gain a better understanding of this process.

### Definitions

For the purposes of this review, the following definitions will be used.

#### Emergency medical services and pre-hospital care

EMS will be defined in this review as ‘the system that organises all aspects of care provided to patients in the prehospital or out-of-hospital environment’ (Mehmood et al., 2018). EMS may be referred to in terms of an individual EMS organisation, or a national, regional or system-wide approach. Pre-hospital care will refer to the ‘well-established branch of medicine, now practised by a broad range of practitioners from first aiders, paramedics, doctors, nurses, first responders, voluntary aid workers and remote medics’, within the out-of-hospital setting (Royal College of Surgeons of Edinburgh, 2021).

#### Policy

Policy is defined as ‘a set of ideas or plans used as a basis for making decisions’ (Collins Dictionary, 2020). Policies are considered to be authoritative documents that are used across different sectors including healthcare, business, education and politics (Patton et al., 2015).

#### Policymaking

Policymaking in healthcare is often described as a complex process (Cairney & Oliver, 2017). Cairney (2013) describes the policymaking process as a policy cycle that combines six stages: (1) agenda setting, (2) policy formulation, (3) legitimation, (4) implementation, (5) evaluation and (6) policy maintenance, succession or success (Cairney, 2019, p. 23).

#### Guidelines

Guidelines are defined as ‘information intended to advise people on how something should be done or what something should be’ (Cambridge Dictionary, 2020c). The term ‘clinical practice guidelines’ specifically refers to ‘statements that include recommendations, intended to optimise patients’ care, that are informed by the systematic review of evidence and an assessment of benefits and harms of alternative care options’ (American Academy of Family Physicians, 2021).

#### Protocols

The term protocol describes ‘the exact method for giving medical treatment’ (Cambridge Dictionary, 2020d). Treatment protocols provide a step-by-step guide on how to perform a particular treatment or intervention, and they are used in areas of practice such as resuscitation and airway management (New York State Department of Health, 2019).

#### Development and formulation

The term development is defined as the ‘process of coming into existence or of creating something new or more advanced’ (Cambridge Dictionary, 2020a). While the term development may be commonly associated with practice guidelines (Woolf et al., 2012), the term formulation is also used within Cairney’s (2013) policy cycle specifically. Hence, the term formulate is defined as ‘to develop all of the details of a plan for doing something’ (Cambridge Dictionary, 2020b).

## Methods

A scoping review is proposed in order to identify the range, nature and extent of the literature available on this topic (Arksey & O’Malley, 2005).

### Protocol design

This scoping review will follow the Arksey and O’Malley (2005) five-stage methodological framework for scoping reviews and adhere to the Preferred Reporting Items for Systematic Reviews and Meta-Analyses extension for Scoping Reviews (PRISMA-ScR) checklist (Tricco et al., 2018).

### Stage 1: the research question

#### Research question

What is known from the existing literature about the development of policy, clinical practice guidelines and protocols within emergency medical services?

#### Aim

The aim of this scoping review is to identify and map the existing evidence on the development of policy, CPG and protocols within EMS. It expects to provide a synthesis of the range, extent and quality of evidence available on this subject and describe any gaps in the existing evidence.

#### Objectives

The main objectives of this review are:

1. To identify and map the existing evidence on the development of policy, guidelines or protocols in EMS using a structured and systematic scoping review.
2. To describe the nature, range and quality of evidence available.
3. To identify and outline any knowledge gaps in the available literature on this topic.

### Stage 2: identification of relevant studies

#### Information sources

This scoping review will involve a broad search for literature relating to the research question and its objectives. It will search key electronic bibliographical databases Medline, CINAHL, Academic Search Complete and PsycINFO via EBSCO, EMBASE via Ovid, Nursing and Allied Health via ProQuest, the Cochrane library and NICE Evidence. It will search for relevant literature using Scopus, OpenGrey repository, British Library E-Theses Online Service (EThOS) and key EMS journals and websites following discussion with experts within the review team. In addition, Google Scholar will be searched to the first 300 search results (Haddaway et al., 2015) and the first 12 pages of results of a Google search included, being performed outside of a personal Google account to try and mitigate any bubble effect or selection bias (Coleman et al., 2020; Curkovic & Kosec, 2018). A date filter will be applied from 2002 to 2021, which coincides with the introduction of EMS guidelines within the United Kingdom and the United States (Institute of Medicine, 2007; JRCALC, 2020).

Key EMS journals and websites will be searched to find any relevant articles that may not be discovered from searching electronic databases due to issues with indexing, databases being incomplete or regional bias (Arskey and O’Malley, 2005). After discussion with expert members within the review team, this review will search: *British Paramedic Journal*, *Journal of Paramedic Practice and International Paramedic Practice*, *Irish Journal of Paramedicine*, *Prehospital Emergency Care*, *Australasian Journal of Paramedicine*, *Canadian Paramedicine*, Paramedic PhD website, Guidelines International Network website and Prehospital Guidelines Consortium website.

#### Search terms

Electronic databases will be systematically searched using relevant key words, indexed terms and proximity and Boolean operators. The review team has utilised the Library and Knowledge Service for NHS Ambulance Services in England (LKS ASE) guidance documents for how to conduct an effective literature search for EMS literature (Holland, 2020) in the planning of the searches. The search used in the CINAHL database (on 10 October 2020) via EBSCO is shown in Table 1.

**Table 1.** Search terms, proximity and Boolean operator combinations used to search the CINAHL database via EBSCO.

**Concept**	**Search number**	**Search terms**
**Context and concept**	S1	(MH practice guideline* N3 Develop*) or (MH practice guideline* N3 implement*) or (MH practice guideline* N3 formulat*) or (MH practice guideline* N3 produc*)
	S2	(Protocol* N3 Develop*) or (protocol* N3 implement*) or (protocol* N3 formulat*) or (protocol* N3 produc*)
	S3	(policy N3 Develop*) or (policy N3 implement*) or (policy N3 formulat*) or (Policy N3 produc*) or (policy N1 making)
	S4	S1 OR S2 OR S3
**Population**	S5	Prehospital OR MH ‘Prehospital Care’
	S6	ems or MH ‘Emergency Medical Services’
	S7	Ambulance* OR ‘ambulance service*’
	S8	Paramedic* OR MH emergency medical technician
	S9	S5 OR S6 OR S7 OR S8
	**Sum**	**S4 AND S9**

MH = CINAHL subject heading; N3 = EBSCO proximity search three words apart.

#### Screening

The results of each database search will be downloaded into EndNote^X9^ reference management software (Clarivate Analytics, 2021) to allow for the efficient removal of duplicate records. The remaining results will be screened for their eligibility for full-text review based on their titles and abstracts. JR will undertake the initial screen and a sample of at least 100 results will be independently screened by a second reviewer to allow for validation of the screening process. Any disagreements or uncertainties will be discussed with a third reviewer (TQ/MH) to come to a consensus.

### Stage 3: study selection

The reviewers will assess the remaining results for eligibility to be included in the review. JR will perform a full-text review of each result and assess its eligibility to be included in the review against the pre-established inclusion and exclusion criteria. A second researcher will independently assess a 10% sample of results to allow for validation of this assessment. Any disagreements or discrepancies will be discussed with a third researcher (TQ/MH) to come to a decision as to whether to include or exclude the results from the review. The reference list and forward citations of eligible articles will be searched to locate any additional material not discoverable in the initial search (Wright et al., 2014).

#### Inclusion criteria

This scoping review will include articles and results that meet the following inclusion criteria:

- **Population and context:** This scoping review will include sources that focus on the people who are involved in the development of policy, CPG or protocols within EMS or pre-hospital care. Therefore, it will include sources of information relating to paramedics, emergency medical technicians, policymakers, actors or stakeholders who take part in this process.
- **Concept:** This review will include sources that include the development, formulation or production of policy, guidelines and protocols. Therefore, it will consider sources that discuss the development process as part of a larger discussion of implementation, however this will not include sources that consider the implementation without the development process. It will include sources that focus upon the development of organisational, regional and national documents owing to the variation in the size and scope of EMS across the world.
- **Types of evidence:** This scoping review will consider a broad range of different sources owing to the relative paucity of empirical research on this topic. It will include primary and secondary research of any methodological tradition, commentary pieces, expert opinions, published conference abstracts, editorials, letters or published narratives as this may be the best available literature on this topic.
- **Language:** Articles that are not published in English will be translated using the Google translate function (Balk et al., 2013).

#### Exclusion criteria

The exclusion criteria are:

- **Population and context:** This review will not consider sources that focus upon in-hospital care or areas of practice outside of EMS or the pre-hospital setting. It will exclude disaster medicine and emergency preparedness, austere environments, events medicine, first aid, military defence forces and tactical combat casualty care or inter-hospital transfers which fall outside of the context of this review. It will also not include articles that do not mention EMS, pre-hospital care or paramedicine specifically.
- **Concept:** This review will not include sources that solely focus upon the utilisation of policy, guidelines and protocols by EMS clinicians at the point of care, or results that focus on implementation of a particular policy, document or guideline if it does not describe the development process.
- **Types of evidence:** This review will not include policy, CPG or protocol documents published by regional EMS organisations to direct their operations or clinical care. While this might include some relevant information, the purpose of this review is to study the existing literature on this process, and not to analyse the numerous internal documents from EMS systems around the world. Posts on social media or EMS organisational websites will also be excluded.
- **Language:** This review will exclude articles that are not available in English and that cannot be translated using Google translate.

### Stage 4: extracting and charting the data

Data will be extracted from eligible studies regarding (a) general information about the results citation and (b) key data relating to the research question and its objectives. Key data will be extracted using a data extraction form adapted from the Joanna Briggs Institute (Peters et al., 2020) guidance for scoping reviews. It will extract data relating to the following areas:

- evidence source, details and characteristics;
- citation details, including: author names, date, title, journal, volume, issue and pages);
- country of origin;
- context/setting;
- method of study;
- results extracted from source of evidence;
- **population** involved: who was involved and what was their role?;
- **concept**: type of document: policy, protocol or guidelines;
- **concept**: how was the development process described? What key actions were involved? What influenced this process?;
- describe the **context**: area of practice;
- summary of findings/results;
- assessment of quality: using the relevant critical appraisal tool; and
- limitations.

Charting will be used to sort primary search results according to their key issues and themes (Arksey & O’Malley, 2005).

### Stage 5: collating, summarising and reporting the results

Data will be charted in tabular form with accompanying narrative descriptions. A table of results will provide an overview of how policy, guidelines and protocol are developed within EMS and the quality of evidence found, and describe any gaps in existing evidence. Although this scoping review will not undertake a structured narrative synthesis, prominent narratives will be iteratively grouped into discoverable themes to allow for discussion.

#### Critical appraisal

The Arksey and O’Malley (2005) framework for scoping reviews does not include an assessment of the quality of evidence. Since the development of this framework in 2005, there has been advancement in the methods and tools available to assess the quality of qualitative studies, CPG and the strength of their recommendations (Brouwers et al., 2010; Centre for Evidence-Based Medicine, 2020), and, where possible, the review team will carry out a critical appraisal of eligible studies, using relevant tools.

#### Dissemination

This review will present an overview of all of the evidence found to inform policymakers, researchers and readers about the range of evidence available on that particular topic. Findings from this scoping review will be submitted for publication. Results will be shared through presentations at scientific and professional meetings, and through social media platforms. As all the researchers hold positions within academia within the United Kingdom, there is also an opportunity for this information to be communicated to undergraduate and postgraduate paramedic students during their academic studies. Finally, as this scoping review forms part of a PhD programme, it will form the scientific basis for further original research into policy, guidelines and protocols within EMS.

## Acknowledgements

The research team would like to thank Darren Flynn from Coventry University, and Matt Holland from the LKS ASE, for their support in developing a search strategy.

## Author contributions

JR, MH, TQ: conceptualisation and design; JR: original draft; MH, TQ: writing and editing. JR acts as the guarantor for this article.

## Conflict of interest

None declared.

## Funding

None.
